# Teaching-learning and patient care

**DOI:** 10.4103/0019-5413.43404

**Published:** 2008

**Authors:** Amit Agrawal

**Affiliations:** *Department of Surgery, Datta Meghe Institute of Medical Sciences, Sawangi, Meghe, Wardha, India*

Sir,

I read the articles with great interest in which the authors have identified the very pertinent issues, particularly in today's perspective, such as teaching–learning,^1^ and postgraduate education including research are well discussed.^2^,^3^ Although the articles have been written in reference to orthopedics and identified problems and potential solutions related to this specialty, after reading these articles carefully, I realized that the questions raised and facts provided are common to many other medical fields and have wider implications. None of us can deny the fact that the education is well organized in United States and Europe, and we are in an advantageous position to have seen the natural history of education in the West.^1^ However, we need to be careful because the clinical problems of the developing countries are diagonally different from those of the developed nations, and it is a very pertinent suggestion to develop strategies best suited for our patient population in their social milieu.^4^ It is beyond doubt that the developing countries will have to work hard and in multiple directions to generate evidence for improving patient care with available infrastructure and to keep pace with the West in upgrading education.^2^ At the same time, we should remember that, at the end, we should be able to translate research into low-cost treatment options without compromising the aims of clinical research, as the cost will always be a concern whatever may be the earning or affordability of the society. With the availability of better investigation modalities and treatment options today, it is possible to draw better conclusions on the past practices.^1^ At the same time, I wish to recognize the efforts of all senior colleagues, because their efforts and hard work have made the learning from the past experiences, continuous evolution of medical science, and dissemination of the knowledge far and wide look so easy. I believe that, by and large, we need to identify a common starting point from where we can start and the distance where we can walk together to help reduce the early exhaustion of resources and to help serve the mankind better.
